# Dissecting the impact of dietary fiber type on atherosclerosis in mice colonized with different gut microbial communities

**DOI:** 10.1038/s41522-023-00402-7

**Published:** 2023-06-03

**Authors:** Evan R. Hutchison, Kazuyuki Kasahara, Qijun Zhang, Eugenio I. Vivas, Tzu-Wen L. Cross, Federico E. Rey

**Affiliations:** 1 Microbiology Doctoral Training Program, University of Wisconsin-Madison, Madison, WI USA; 2 Department of Bacteriology, University of Wisconsin-Madison, Madison, WI USA; 3 Lee Kong Chian School of Medicine, Nanyang Technological University, Singapore, Singapore; 4 Department of Nutrition Science, Purdue University, West Lafayette, IN USA

**Keywords:** Microbiome, Microbiota, Metagenomics

## Abstract

Dietary fiber consumption has been linked with improved cardiometabolic health, however, human studies have reported large interindividual variations in the observed benefits. We tested whether the effects of dietary fiber on atherosclerosis are influenced by the gut microbiome. We colonized germ-free *ApoE*^−/−^ mice with fecal samples from three human donors (DonA, DonB, and DonC) and fed them diets supplemented with either a mix of 5 fermentable fibers (FF) or non-fermentable cellulose control (CC) diet. We found that DonA-colonized mice had reduced atherosclerosis burden with FF feeding compared to their CC-fed counterparts, whereas the type of fiber did not affect atherosclerosis in mice colonized with microbiota from the other donors. Microbial shifts associated with FF feeding in DonA mice were characterized by higher relative abundances of butyrate-producing taxa, higher butyrate levels, and enrichment of genes involved in synthesis of B vitamins. Our results suggest that atheroprotection in response to FF is not universal and is influenced by the gut microbiome.

## Introduction

Individual responses to the same diet or therapeutic drugs are often inconsistent and not universal. This notion is a fundamental principle of precision medicine and nutrition^1,2^. Many factors influence how a subject responds to a given treatment including genetics, diet, and sex. Recently, it has become apparent that the gut microbiome is a major contributor to the observed interpersonal variation in responsiveness^3–6^. It is now widely recognized that the gut microbiome plays a significant role in health and its composition is highly variable among individuals^7^. Dietary components, from foodstuffs to orally administered drugs, come in close contact with resident microbes along the gastrointestinal tract. The gut microbiome collectively encodes >100-fold more genes than the human genome, including a rich array of enzymes with the potential to metabolize these ingested compounds and modulate their bioavailability, activity, and ultimately their effects on the host^8–10^. Indeed, gut microbes have received considerable attention in recent years for their capacity to modulate responses to bioactive compounds^11^ ranging from antihypertensive drugs to immunosuppressants for organ transplants^12,13^. Gaining a better understanding of which interventions are most sensitive to microbiome variation is critical for the effective implementation of precision medicine.

Cardiovascular disease (CVD) is the leading cause of death in the United States and accounts for over a third of all deaths globally^14,15^. Atherosclerosis is the most common manifestation of CVD and is driven by inflammatory processes that result in the formation of macrophage-dense, fatty plaques within the arterial wall^16^. There is increasing evidence that the gut microbiome plays an important role in modulating atherosclerosis development. Epidemiological studies have identified differences in the microbiomes of individuals with coronary artery disease compared to healthy individuals^17–19^. Furthermore, several microbial metabolites arising from specific dietary components have been shown to modulate atherosclerosis progression in humans and animal models through a variety of mechanisms. For example, trimethylamine *N*-oxide, a microbial derivative of choline, is associated with increased risk of major cardiovascular events in humans^20^; the microbial metabolite indole-3-propionic acid, which is derived from tryptophan, protects against atherosclerosis progression by promoting cholesterol efflux^21^; and short-chain fatty acids (SCFAs), which are produced via fermentation of dietary fiber, have been shown to ameliorate atherosclerosis by limiting dietary cholesterol absorption (propionate) and reducing inflammation and gut permeability (butyrate)^22–24^. Indeed, diet has long been known to play a major role in both the promotion and prevention of atherosclerosis^25,26^. For example, it is well-established that foods such as whole-grain cereals and legumes which are rich in dietary fiber, are protective against CVD^27,28^. However, inconsistent responses to a number of dietary and pharmacological interventions for CVD have been observed between individuals^29,30^. Most studies linking dietary fiber to improved cardiovascular health are assessed using population averages^31^ and do not account for individual characteristics. Therefore, the causes behind these inconsistencies are understudied.

Dietary fibers are oligo- or polysaccharides that resist degradation by host enzymes and are available to be metabolized by microbes in the distal gut. Dietary fibers vary widely in structure and composition and are often subdivided according to their biochemical properties. One such division is drawn by whether they can be fermented by gut microbes. Thus, fermentable fibers are dietary fibers that can be metabolized by intestinal microbes, while non-fermentable fibers resist intestinal fermentation^32^. By this definition, fermentability is not a static or inherent property of any given fiber since it is context-dependent and is contingent on the presence of specific microbes that can degrade the fiber in question and the host environment (e.g., transit time, rumination). Fermentation of dietary fiber in the gastrointestinal tract results in the production of SCFAs, the most abundant of which are acetate, propionate, and butyrate. SCFAs have been linked to improved cardiometabolic health^22,23^ and are hypothesized to mediate some of the atheroprotective effects associated with dietary fiber consumption^33^. However, multiple studies have shown that SCFA production is dependent on microbiome structure and is highly variable among individuals. For example, McOrist et al. found individualized responses in butyrate production to a resistant starch (RS) dietary supplement^34^. Additionally, we recently found that various fermentable fibers (pectin, inulin, fructo-oligosaccharide (FOS), RS-2, and RS-4) elicited disparate responses in SCFA production when fed to mice colonized with different microbial communities^35^.

Given the personalized nature of the gut microbiome and the fact that gut microbes are necessary for metabolizing dietary fiber, we hypothesized that the atheroprotective effects attained in response to a given dietary fiber are modulated by the gut microbiome composition of the consumer. To test this, we colonized groups of germ-free (GF) *apolipoprotein E* deficient (*ApoE*^−/−^) mice with fecal microbial communities from one of three human donors, each of which exhibited divergent microbial compositions and SCFA-producing capacities. Colonized mice were fed a diet containing either a mixture of fermentable fibers (FF) or a non-fermentable cellulose control diet (CC). We found that protection from atherosclerosis by FF consumption was not universal, but was instead modulated by the resident microbiome. We also observed that atheroprotection was associated with increased butyrate production and enrichment for bacterial genes involved in pathways for carbohydrate metabolism and vitamin synthesis.

## Results and discussion

### Engraftment of donor communities prior to dietary treatment

Germ-free (GF) female *ApoE*^−/−^ mice were colonized with fecal samples from one of three human donors (Supplementary Fig. 1). These samples were selected from a repository of fecal specimens previously collected from adults in their mid-seventies^36^ and were chosen based on (i) their divergent community structure as assessed by unweighted UniFrac distances of 16S rRNA profiles and (ii) their capacity to generate differing levels of SCFAs when engrafted in GF mice consuming a semi-purified diet containing an assortment of fibers^35^. After colonization, mice were maintained on the FF diet for two weeks to allow for stabilization of engrafted communities before the dietary treatment phase (Supplementary Fig. 1). Fecal samples were collected at this point to assess bacterial engraftment via 16S rRNA V4 amplicon sequencing. The engraftment efficiency (genus level) was 64% in DonA-, 65% DonB-, and 72% DonC-colonized mice, respectively (Supplementary Fig. 2f). When calculated as the percentage of donor genera detected in at least one of the recipient mice, efficiencies were 90, 88, and 87% in DonA-, DonB-, and DonC-colonized mice, respectively (Supplementary Fig. 2c–e). These engraftment efficiencies are in line with previous studies^37,38^. Physiological, anatomical, and behavioral differences between human donors and recipient mice along with differences in diet likely explain why only a fraction of the donor bacteria engrafted.

A few genera were only detected in the recipient mice but were unique to each of the three donor groups, suggesting that these taxa may have been present in low abundance in the human donor samples rather than the result of contamination. Principal coordinate analysis (PCoA) of weighted UniFrac distances of fecal samples collected prior to dietary treatment showed uniform engraftment between mice bound for the two diets (FF-bound and CC-bound) within all treatment groups (all pairwise PERMANOVA adjusted *P* > 0.1, Supplementary Fig. 2a). However, comparisons using unweighted UniFrac distances (sensitive to presence/absence of taxa) showed a significant difference in community structure in DonA-colonized mice between FF-bound and CC-bound communities (adjusted *P* = 0.0012, Supplementary Fig. 2b). This was driven by 9 genera that were detected in one diet-bound group but not the other (Supplementary Fig. 2c). Eleven weeks after dietary treatment, cecal samples were collected and used to assess terminal microbial communities. By the end of the experiment, 5 of the 9 missing genera were no longer detected in cecal contents of mice on either diet, while 4 genera (*Clostridium*, *Faecalibacterium*, *Gemmiger*, and an undetermined *Ruminococcus* genus) were found only in FF-fed mice (Supplementary Fig. 2c). This introduces the possibility that the differences observed in the assembled communities between dietary groups for DonA mice are the result of inconsistent engraftment rather than an effect of diet. Alternatively, since microbial communities undergo considerable fluctuations in the period after colonization^39^, it is possible that these missing taxa were present in the CC-bound mice, but below detectable levels. The latter scenario is supported by the fact that (i) all of the missing taxa were detected in the human donor sample used to inoculate all DonA mice, and (ii) similar FF-diet-driven patterns were observed with *Faecalibacterium* and *Gemmiger* abundances in a previous study^35^ that used the same donor feces and the same diets. These findings highlight the importance of reporting pre-treatment engraftment data in mouse transplant studies such that the conclusions can be appropriately contextualized.

### Diet-induced shifts in microbiota composition are largely community-specific

**Fig. 1:**
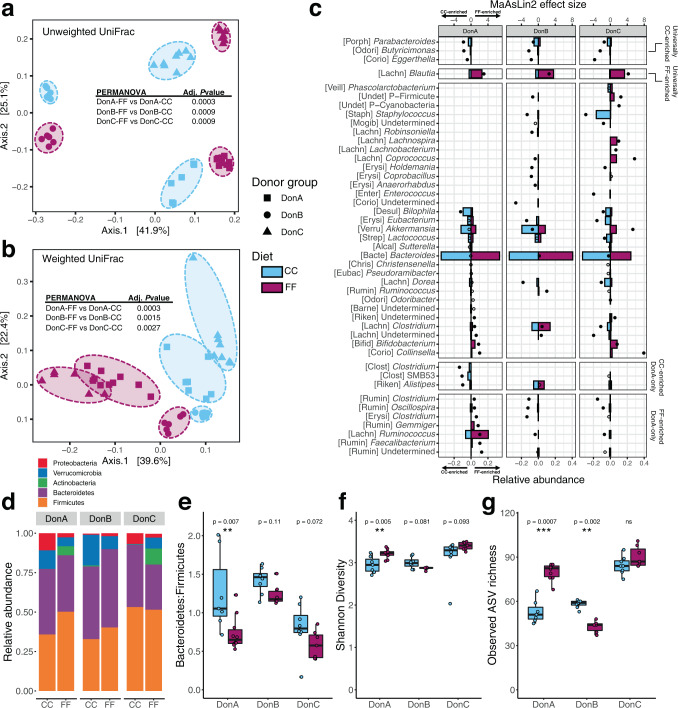
Effects of dietary fiber on microbial community structure in gnotobiotic mice colonized with different human communities. **a**, **b** Principal Coordinate Analysis of unweighted and weighted UniFrac distances of 16S rRNA V4 ASVs. Animals colonized with fecal samples from three different donors: DonA, DonB, and DonC. **c** Relative abundances of genus-level taxa for the two diets (bottom *x*-axis, colored bars) along with between-diet differential abundance (MaAsLin 2) effect sizes (top *x*-axis, dots). Significant differences in abundance for taxa within each donor group are denoted by a solid dot (adjusted *P* < 0.1) and open circles denote no significant change (adjusted *P* > 0.1). The dot’s orientation relative to the origin represents the effect of diet on the abundance of each taxa (negative values correspond to CC abundances, positive values correspond to FF abundances). The first 5 letters of the family encompassing each taxon is shown in brackets; if the family is undetermined the taxon phylum is listed instead and noted with a “P-”. **d** Relative abundance of Phylum-level taxa as a function of diet and donor group. **e** Bacteroidetes to Firmicutes ratio. **f**, **g** Shannon diversity index and observed richness. Box and whisker plots denote the interquartile range, median, and spread of points within 1.5 times the interquartile range along with individual data points; magenta = Fermentable Fiber (FF), blue = Cellulose Control (CC). Comparisons of means (*n* = 7–10/diet/donor group) conducted with Wilcoxon test, *P < 0.05, **P < 0.01, ***P < 0.001.

Two weeks after colonization, mice were placed into their dietary treatment groups and were maintained on their respective diets for 11 weeks. PCoA analysis of weighted and unweighted UniFrac distances (Fig. 1a, b) of cecal bacterial communities assessed at the completion of the study shows that mice were highly distinguishable by dietary treatment within each donor group. When using unweighted UniFrac distances, ordination shows that donor group had a stronger effect on community composition than diet (Fig. 1a). PCoA of weighted UniFrac distances, which factors in the abundance of each taxon, shows clear distinctions by diet, but more overlap between donor groups (Fig. 1b), suggesting that FF consumption elicits distinct changes of some low-abundance, phylogenetically-related taxa across the three communities. Alpha diversity (Shannon) was higher in mice colonized with DonA consuming FF relative to CC consumption but was not significantly impacted by diet in DonB- or DonC-colonized mice (Fig. 1f). Similarly, observed amplicon sequence variant (ASV) richness was significantly increased with the FF diet in DonA mice but was reduced by FF feeding in DonB mice (Fig. 1g). FF-consumption resulted in increased DNA concentration in cecal content, a proxy for microbial biomass^40^, compared to CC-fed mice (Supplementary Fig. 3). This effect was observed in all three donor groups and suggests that a diet rich in fermentable fiber generally increases microbial biomass.

Bacterial communities were dominated by Firmicutes and Bacteroidetes and had detectable levels of Proteobacteria, and Verrucomicorbia in all treatment groups (Fig. 1d). Actinobacteria were detected in FF-fed DonA-colonized mice, CC-fed DonB-colonized mice, and both diets for DonC-colonized mice. FF feeding lowered the Bacteroidetes to Firmicutes ratio for all donor groups (Fig. 1e), but the only significant reduction was observed in DonA-colonized mice. There was generally little consistency in the diet-associated enrichment patterns observed across donor groups, even when considering phylum level changes, suggesting a lack of a universal response to dietary treatment (Fig. 1c–g).

We used the Microbiome Multivariable Association with Linear Models (MaAsLin 2)^41^ to analyze enrichment patterns at the genus level caused by diet for each donor group. Most genera that were significantly different (adjusted *P* < 0.1) were unique to each donor group: *Blautia* was the only genus that was significantly enriched by FF-consumption across all donor groups, while *Eggerthella, Butyricimonas*, and *Parabacteroides* were the only genera to exhibit universal enrichment in response to the CC diet (Fig. 1c). One obvious explanation for the lack of universality is the fact that each donor group possess different collections of microbes. However, even in the cases of genera that were present across multiple donors, the directionality in response to diet tended to vary by community ([Lachn] *Clostridium*, *Bacteroides*, *Akkermansia*, [Lachn] Undetermined, [Rumin] *Clostridium*, *Oscillospira*, [Erysi] *Clostridium*, [Lachn] *Ruminococcus*). For example, the *Bacteroides* genus was significantly enriched by CC feeding in DonC-colonized mice, by FF feeding in DonB-colonized mice and unaffected by diet in DonA-colonized animals (Fig. 1c). Interestingly, the relative abundance of *Akkermansia* was significantly increased by CC feeding in DonB mice, by the FF diet in DonC mice, and was not affected by diet in DonA-colonized mice. *Akkermansia muciniphila*, the most prevalent species in this genus, feeds on host mucins which can be glycosylated by fiber-degrading bacteria thereby influencing *Akkermansia* levels^42,43^. Although *Akkermansia* has been reported to thrive on diets poor in fermentable fiber^44^, our data indicate that microbiota composition can influence *Akkermansia* response to specific fiber sources. It is also possible that the different donor groups possess distinct strains of *Akkermansia muciniphilia* that are themselves differentially affected by diet. Together, these results suggest that microbial responses to dietary fiber are context-dependent and are likely impacted by the composition and metabolic capabilities of the broader community.

### Fermentable fiber impacts atherosclerosis progression in a donor-dependent manner

**Fig. 2:**
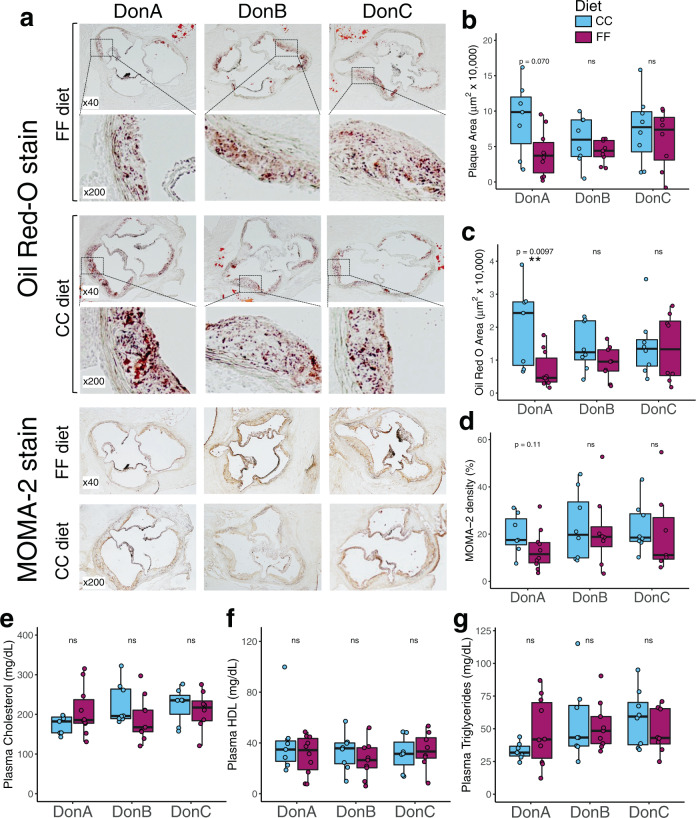
Atherosclerosis response to dietary fiber in mice colonized with different human communities. Atherosclerosis was measured in GF *ApoE*^−/−^ mice colonized with human fecal communities DonA, DonB, and DonC and fed either fermentable fiber diet, or a cellulose control diet. **a** Representative Oil Red O staining and MOMA-2 antibody staining of aortic sinus cross-sections content. Quantification of plaque average area (**b**), lipid positive area (**c**) or MOMA-2 positive area (**d**). Plasma levels of total cholesterol (**e**), HDL-cholesterol (**f**) and triglycerides (**g**). Box and whisker plots denote the interquartile range, median, and spread of points within 1.5 times the interquartile range along with individual data points; magenta = Fermentable Fiber (FF), blue = Cellulose Control (CC). Comparisons of means between diets within each donor group (*n* = 7–10/diet/donor group) were conducted using a Wilcoxon test with appropriate correction for equal variance assumption (Levenes’ test), *P < 0.05, **P < 0.01.

FF-fed mice colonized with DonA had significantly reduced lipid deposition in atherosclerotic plaques and a trend towards reduced plaque area (*P* = 0.070) compared to their CC-fed counterparts, while there were no differences observed between diets in either DonB- or DonC-colonized mice (Fig. 2a–c). To further characterize atherosclerosis disease status, lesions were assessed for macrophage infiltration by immunohistology with MOMA-2 antibodies. There were no statistically significant differences observed between diets in lesion MOMA-2 density in any of the donor groups (Fig. 2a, d), but there was a trend of reduced density with FF feeding in DonA mice (*P* = 0.11). Previous studies report inconsistent results regarding of the effect of inulin (a component fiber in the FF diet) on atherosclerosis in mice^45,46^. Rault-Nania et al. found that inulin ameliorated atherosclerosis in *ApoE*^−/−^ mice, whereas Hoving and colleagues found that inulin exacerbated atherosclerosis in APOE*3-Leiden mice. While this discrepancy could be due to the different diets and/or mouse models used in these studies, our results support the notion that the gut microbiome modulates the atheroprotective effect of fermentable dietary fiber, providing a possible explanation for these conflicting findings.

### The atheroprotective effect of the FF diet is not associated with changes in plasma lipids or alterations in the expression of aortic immune markers

To test whether fermentable fiber consumption altered lipid composition in circulation, we measured lipid levels in the plasma of the mice described above. No statistical differences were observed between diets for any of the donor groups in plasma levels of total cholesterol, HDL cholesterol, or triglycerides (Fig. 2e–g). We also assessed aortic expression levels of *Abca1* and *Abcg1* mRNA by RT-qPCR as markers of reverse cholesterol transport but did not observe any statistically significant differences between diets within any of the donor groups (Supplementary Fig. 4d, e). These results suggest that the atheroprotective effect of FF consumption observed in DonA mice was not mediated by major alterations in plasma lipids or cholesterol homeostasis. To test whether atheroprotection was associated with changes in vascular inflammation status, we measured aortic expression of the inflammatory markers *Tnf*-*α*, *Il1*-*β*, and *Vcam-1*, which are commonly associated with atherosclerosis progression^22,47^. There were no significant differences observed in expression of these markers between diets in any of the donor groups (Supplementary Fig. 4a–c). These data suggest that the atheroprotective effect of FF-feeding in DonA mice may be independent of these immune processes and reverse cholesterol transport, although further analyses are needed to fully rule out these factors.

### Cecal SCFA profiles are altered by diet in a donor-dependent manner

**Fig. 3:**
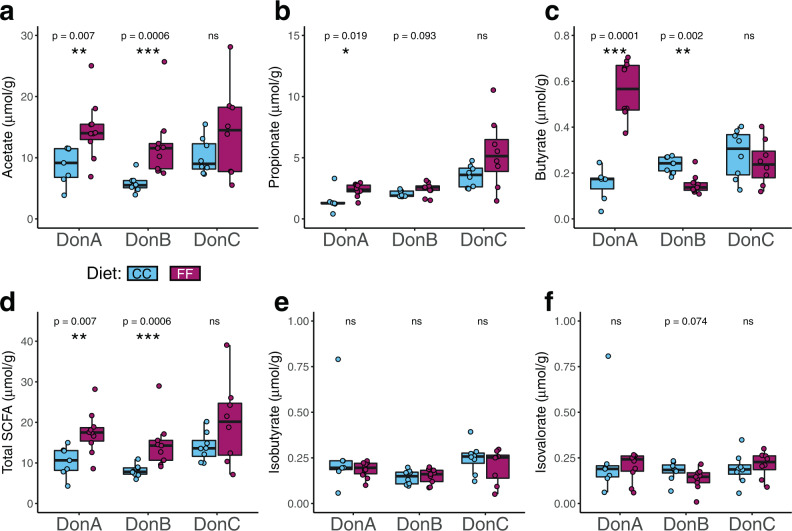
Effect of dietary fiber on cecal levels of SCFAs and branched-chain fatty acids. Cecal levels of acetate (**a**), propionate (**b**), butyrate (**c**), total SCFAs (sum of acetate, propionate, and butyrate) (**d**), isobutyrate (**e**) and isovalerate (**f**). Concentrations are expressed per gram of cecal content wet weight. Box and whisker plots denote the interquartile range, median, and spread of points within 1.5 times the interquartile range along with individual data points. Comparisons of means between diets within each donor group (*n* = 7–10/diet/donor group) were conducted using a Wilcoxon test, *P < 0.05, **P < 0.01, ***P < 0.001.

Given the variability in microbiome composition between diets, we next tested whether there were differences in fiber fermentation capacities between donor groups. In line with the microbiome composition patterns above, diet-induced shifts in SCFA profiles were highly dependent on donor group (Fig. 3a–d). Acetate, the most abundant SCFA, was increased in FF-fed mice colonized with both DonA and DonB communities (Fig. 3a). Propionate was increased by FF-feeding only in DonA mice, whereas diet did not affect propionate levels in the other two donor groups (Fig. 3b). FF-feeding resulted in substantially elevated cecal butyrate levels in DonA mice, but reduced butyrate levels in DonB-colonized mice compared to CC-fed counterparts (Fig. 3c). Cecal butyrate concentrations were not different between diets in DonC-colonized mice. The branched-chain fatty acids isobutyrate and isovalerate, which are primarily produced via protein fermentation, were not affected by diet within any of the donor groups (Fig. 3e, f). A lack of differences in branched-chain fatty acids is consistent with the fact that the FF and CC diets are isoproteic. Total SCFA concentrations (i.e., the sum of acetate, propionate, and butyrate) within donor groups were increased by FF-feeding in DonA and DonB, but not DonC (Fig. 3d). These results reflect the shifts observed in SCFA-producing microbiota. Among the genera that were increased by FF feeding in DonA mice only were *Clostridium*, *Oscillospira*, *Ruminococcus*, *Gemmiger*, and *Faecalibacterium*, (Fig. 1c) all of which contain butyrate-producing species^48,49^. Notably, most of these genera were also present in the other donor groups but were not enriched by FF-feeding. This could be due to complex, community-level interactions (e.g., competition) influencing responses of individual genera to dietary fiber, or strain-level differences in response to diet, or both.

Our data are consistent with previously reported studies suggesting that butyrate-induced protection against atherosclerosis in mice occurs in the absence of major changes in plasma lipid levels^22,24^. Similar to our results, Kasahara et al.^22^ reported that the introduction of a butyrate-producing microbe in mice colonized with a simplified bacterial community led to reductions in plaque burden and macrophage infiltration in *ApoE*^−/−^ mice without significant changes in cholesterol homeostasis. However, Kasahara et al. also detected butyrate-induced reductions in aortic expression of inflammatory markers *Tnf*-*α*, *Il1*-*β*, and *Vcam-1*, which we did not observe in our study. Additionally, a recent study found that propionate consumption protected against atherosclerosis by inhibiting cholesterol uptake in the intestine^23^. Although we observed increased cecal propionate levels in DonA-colonized mice consuming the FF diet, we did not detect differences in plasma cholesterol levels. The concentration and site within the gastrointestinal tract where propionate accumulates (i.e., greater concentration in the small intestine when consumed orally vs. greater concentration in the large intestine when produced via fiber fermentation) may influence its effect on cholesterol absorption. Together, these findings suggest that SCFA-production capacity is dependent on both accessible dietary fiber and microbial community composition. These results also validate the notion that the abundance of butyrate-producing microbes is associated with cecal levels of butyrate.

### Bacterial functional profiles were modulated by dietary fiber in a donor-specific manner

**Fig. 4:**
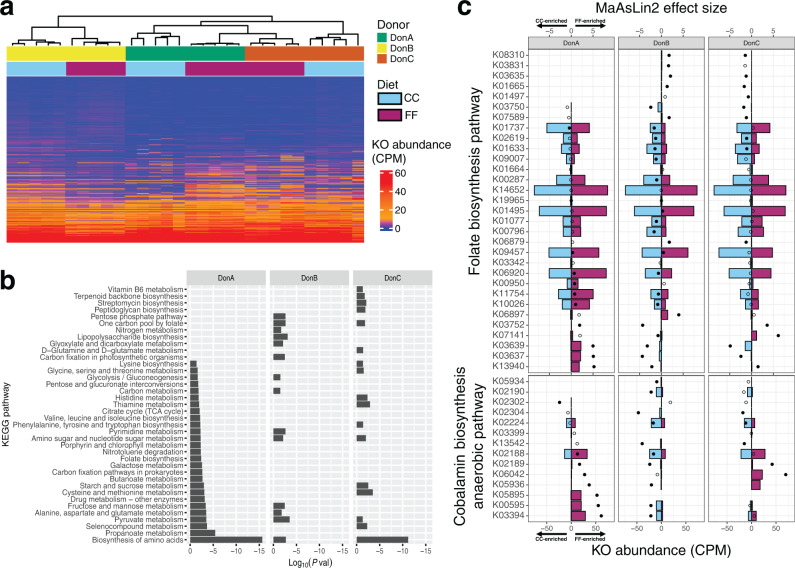
Effect of dietary fiber on KEGG orthology profiles and metagenomic pathway enrichment across donor groups. **a** Heatmap of KO profiles expressed in CPM for each mouse. Individual profiles were clustered using the spearman hierarchical clustering method. **b** Overrepresented KEGG pathways and their significance (represented as the log_10_ of the enrichment *P*-value) identified by pathway enrichment analysis using lists of KOs from each donor group that were significantly upregulated (MaAsLin 2 differential abundance, adjusted *P* < 0.1). **c** MaAsLin 2 differential abundance (bottom axis, colored bars) and effect size (top axis, solid dot = adjusted *P* < 0.1, open dot = adjusted *P* > 0.1) of KOs involved in folate biosynthesis and cobalamin (vitamin B12) biosynthesis. Negative values reflect KO abundance (CPM) in CC-fed mice and effect sizes (MaAsLin 2 coefficient) favoring the CC condition, while positive values indicate KOs abundances and effect sizes in the FF condition (*n* = 5/diet/donor group).

We next sought to identify links between the functional potential of the microbiome and atheroprotection by examining changes in microbial metagenomic profiles. We performed shotgun sequencing of DNA isolated from cecal contents (average of 29.4 ± 7.7 million paired-end reads/sample; *n* = 5/diet-donor group). Sequence data were analyzed with HUMAnN3 to generate metagenomic functional profiles that included KEGG orthology (KO) abundances for each mouse. Hierarchical clustering of KO profiles using Bray–Curtis dissimilarity shows that the treatment groups are different from one another, but the effect of diet on clustering patterns varied by donor (Fig. 4a). Mice colonized with DonA and DonC communities clustered closer by diet than by donor group, suggesting a significant level of FF-influenced overlap in KO profiles between these two donor groups. DonB mice, on the other hand, clustered separately from all other mice but sub-clustered by diet (Fig. 4a). Differential abundances of individual KOs between FF- and CC-fed mice within each donor group were calculated with MaAsLin 2. This analysis revealed that 2676 KOs were significantly different (adjusted *P* < 0.05) between diets in at least one donor group (DonA = 971; DonB = 1964; DonC = 1419). Of these, only 67 (2.5%) were enriched by FF-feeding across all donor groups, whereas 79 (3%) were enriched by CC-feeding across all donor groups, suggesting that most of the diet-induced changes in functional profiles were donor-specific. DonA- and DonC-colonized mice shared the most FF-enriched KOs with 326, while DonA- and DonB-colonized mice shared 99, and animals colonized with DonB- and DonC-colonized mice shared 81 KOs.

We next aimed to gain further insight into how dietary treatment affected the metabolic pathways of the cecal microbial communities in each donor group. We were specifically interested in identifying pathways that might help explain the atheroprotection associated with FF-feeding in DonA. We used the MicrobiomeAnalyst KEGG pathway tool^50^ to conduct pathway enrichment analysis in the KOs that were overrepresented by FF-feeding relative to their counterparts consuming the CC diet. Similar to the taxonomy results discussed above, we observed a lack of universality among the metagenomic changes in response to diet. Of the 38 KEGG pathways that were detected as significantly overrepresented (adjusted *P* < 0.1) by FF-feeding in at least one donor group, only three (*Pyruvate metabolism*, *Amino sugar and nucleotide sugar metabolism*, and *Biosynthesis of amino acids*) were observed across all three donor groups (Fig. 4b). In DonA-colonized mice, 27 pathways were significantly overrepresented in the FF diet relative to CC diet. These included pathways involved in vitamin synthesis (*Thiamine biosynthesis*; *Folate biosynthesis*; *Porphyrin metabolism* [vitamin B12]), SCFA synthesis (*Butanoate metabolism*; *Propionate metabolism*), and amino acid metabolism (*Lysine biosynthesis*; *Cysteine and methionine metabolism*; *Histidine metabolism*; *Phenylalanine, tyrosine and tryptophan biosynthesis*; *Valine, leucine and isoleucine biosynthesis*; *Glycine, serine and threonine metabolism*; *Biosynthesis of amino acids*) (Fig. 4b). Interestingly, the dietary effects on the enrichment of pathways involved the synthesis of acetate (*Carbon metabolism, Glyoxylate and dicarboxylate metabolism*, and *Pyruvate metabolism*), propionate (*Propionate metabolism*), and butyrate (*Butanoate metabolism*) corresponded very closely with the cecal SCFA levels described above (Figs. 4b and 3a–c).

Both folate and vitamin B12 are involved in the detoxification of homocysteine, a metabolite of methionine metabolism that has been linked to cardiovascular disease^51,52^. A study involving *ApoE*^−/−^ mice with hyperhomocysteinemia found that supplementation with a mixture of folate, vitamin B12, and vitamin B6 protected against atherosclerosis^53^. Moreover, a recent metagenomic study in humans showed that patients with CVD (*n* = 218) had decreased abundance of genes encoding for components of the folate biosynthesis pathway than healthy patients (*n* = 187)^17^. Interestingly, the authors of that study also found that CVD was associated with lower abundances of propionate and butyrate synthesis genes. To gain a more detailed picture of the metagenomic dynamics of these pathways, we compared the differential abundances of the individual KOs involved in folate biosynthesis (KEGG map00790) and anaerobic cobalamin (vitamin B12) biosynthesis (KEGG M00924). In agreement with our enrichment analysis, most of the differentially abundant KOs in both pathways were significantly upregulated by FF feeding in DonA-colonized mice, but not in the other groups (Fig. 4c). Folate and vitamin B12 were supplied in the FF and CC diets at the same inclusion rate (AIN-93 vitamin mix, Supplementary Table 1), but it is possible that some amount of additional vitamin availability via microbial biosynthesis may have a physiological effect in host homocysteine metabolism. These results suggest that microbial production of vitamins B12 and folate may act as a potential mediator of the atheroprotection associated with FF diet in mice colonized with this community.

**Fig. 5:**
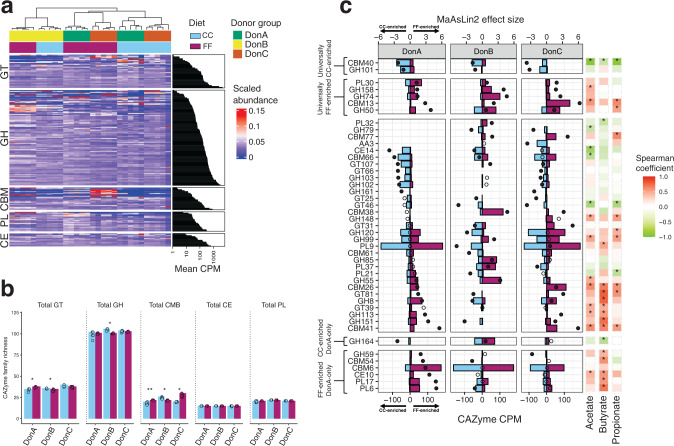
Effect of dietary fermentable fiber on microbial Carbohydrate Active EnZyme (CAZyme) profiles. **a** Heatmap of CAZyme family profiles arranged by category (Glycoside hydrolase = GH; glycosyltransferases = GT; carbohydrate binding modules = CBM; carbohydrate esters = CE; and polysaccharide lyases = PL) along with the mean counts for each CAZyme family expressed in counts-per-million (CPM). Mice profiles were clustered using the Spearman hierarchical clustering method. **b** Comparison of CAZyme family richness between diets (total number of CAZyme families detected within each CAZyme category) by donor group. Barplots denote the mean with individual data points; comparisons of means between dietary groups were conducted using the Wilcoxon test; *P < 0.05, **P < 0.01. **c** MaAsLin 2 differential abundance (bottom axis, colored bars) and effect size (top axis, solid dot = adjusted *P* < 0.1, open dot = adjusted *P* > 0.1) of the top 10% most differentially abundant CAZymes (*n* = 5/diet/donor group). The right panel depicts a heatmap of Spearman correlation coefficients between each corresponding CAZyme family and cecal SCFA levels across all mice, *P < 0.05.

To uncover associations between atheroprotection and fiber metabolism, we determined the level and type of carbohydrate-active enzyme (CAZyme) families between dietary treatments within each donor group using cecal metagenomic data. We detected a number of CAZyme families that were highly abundant in all donor groups and largely unaffected by diet (Fig. 5a, b, Supplementary Fig. 5). Differential abundance analysis revealed that the CAZyme families which were most significantly affected by diet were about 100-fold lower than the highest abundance CAZymes (Fig. 5c). Given the differences in cecal SCFA levels between treatment groups, this suggests that these highly-differential, low-abundance CAZymes have an outsized impact on the dynamics of SCFA metabolism. To highlight the most differentially abundant CAZyme families, we compared the abundances of the CAZyme families that were most affected by diet (top 10% by MaAsLin 2 effect size within each donor group, Fig. 5c). The vast majority of FF-enriched CAZymes in DonA-colonized mice were significantly correlated (Spearman, *P* < 0.05) with cecal butyrate levels, potentially linking them to butyrate production (Fig. 5c). One such CAZyme family, GH59, encompasses β-galactosidases which free terminal β-D-galactose monomers from galactan side chains of pectin^54^. Interestingly, many commonly cited CAZyme families involved in inulin, pectin, RS-2/4, and scFOS were not found among the most highly differentially abundant CAZymes in our dataset. It is possible that the inclusion of multiple fermentable fibers creates competition among microbes that are specialized for each fiber type, reducing the magnitude of changes detected in fiber-specific CAZymes.

In summary, we showed that the gut microbiome regulates the effect of dietary fiber on atherosclerosis development in gnotobiotic *ApoE*^−/−^mice colonized with different human fecal communities. We found that diet-induced shifts in microbial composition, metabolic potential, and metabolic output (SCFAs) varied among the different donor groups. Our results showed that atheroprotection was associated with increased cecal butyrate levels and abundances of butyrate-producing organisms. Additionally, shotgun metagenomic sequencing revealed donor-dependent shifts in genes involved in carbohydrate metabolism, SCFA production, and vitamin synthesis. These data support the notion that diet-associated shifts in the gut microbiota are not solely a function of diet but are instead the result of complex interactions between diet and the larger gut microbial community structure and functional network. These results are also in line with previous work showing that butyrate is atheroprotective without modifying cholesterol metabolism^22,24^.

The current study has some limitations that should be addressed. First, we observed an imperfect engraftment efficiency from human donor to mouse recipient. As discussed above, we detected differences in the pre-treatment engraftment patterns of DonA mice. Our data suggest that this difference in detection was likely a consequence of the stochasticity of microbial communities shortly (two weeks) after colonization and not a result of differences in inoculation or contamination. Nonetheless, this discrepancy introduces the possibility that the differences observed in atherosclerosis within DonA mice were due to inconsistent engraftment rather than response to diet. Another limitation is that our study only used three human donors. A much larger and more diverse cohort of donors would be needed to fully appreciate the breadth of cardiometabolic responses to these diets, but the limited group used here is sufficient to demonstrate that the athero-modulatory effect of dietary fiber is microbiota-dependent. This study is additionally limited by the use of only female mice, precluding us from testing the effect of sex. Finally, the CC and FF diets used in this study differed slightly in their starch content, which may contribute to the differences described above.

Despite these limitations, the work presented here suggests that microbiome variation modulates responses to dietary fiber consumption, which can differentially impact the development of atherosclerosis. Together, these results support the notion that dietary interventions are not universally efficacious and should be tailored to individuals. More research is needed to understand the relevant mechanisms and the metabolic and ecological dynamics that govern the microbiome-dependent individual responses to diet.

## Methods

### Germ-free animals

All animals in the current study were handled and maintained in accordance with the University of Wisconsin–Madison, standards for animal welfare and all protocols were approved by the university’s Animal Care and Use Committee. Germ-free (GF) *ApoE*^−/−^ mice (derived GF from B6.129P2-Apoe^tm1Unc^/J; Jax 002052) were housed in a controlled environment within gnotobiotic isolators under a 12-h light/dark cycle and received autoclaved water and chow (LabDiet 5021; LabDiet, St. Louis, MO) *ad libitum*. Mice were housed with Alpha-dri® (Shepherd Specialty Papers, Kalamazoo, MI) bedding and were enriched with paper huts (Bio-Huts, Bio-Serv, Flemington, NJ) and ALPHA-twist^TM^ (Shepherd Specialty Papers). The GF status of the isolators was evaluated monthly via PCR using universal 16S rRNA primers with fecal DNA as well as a growth test of feces in rich media incubated at 37 °C aerobically and anaerobically for 7 days.

### Selection of human donors

Human fecal samples used in this study were collected from participants as part of the Wisconsin Longitudinal Study (WLS)^36,55^ and stored −80 °C. WLS data and specimen collection were approved by the UW-Madison Internal Review Board (2014-1066, 2015-0955) and written informed consent was obtained in the original study^55^. In a previous publication from our group^35^, a subset of candidate WLS specimens were selected based on their distinct bacterial community structures and then subsequently transplanted into GF mice to measure cecal SCFA profiles. In the current study, we used this information to select three human donor samples: microbiota from donor WLS-sample-8 (referred to here as DonA); donor WLS-sample-1 (referred to here as DonB) and donor WLS-sample-5 (referred to here as DonC). In our previous study^35^, we found that gnotobiotic mice consuming a semi-purified diet containing an assortment of fibers that included resistant starch type 2 and 4, short-chain fructo-oligosaccharides, inulin, and pectin colonized with DonA, DonB or DonC accumulated different levels of SCFA. Mice colonized with DonA accumulated the highest levels of cecal butyrate (~1.5 mM) among all of the donors tested, whereas DonB-colonized mice accumulated significantly lower levels of cecal butyrate (~ 0.6 mM), and mice colonized with DonC showed the highest cecal propionate levels (~10 mM) and intermediate butyrate levels (~1.0 mM)^35^. All of the WLS specimens used in the current study were obtained from subjects that self-reported consuming a western-style diet, were overweight (BMI > 25), and were not diagnosed with diabetes, cancer, or heart disease^35,36^. Identifiable information of WLS participants was blinded to the researchers in the current study.

### Colonization of gnotobiotic mice with human feces and dietary treatment

At 6 weeks of age, mice were transferred to ventilated cages on an Allentown Sentry SPP IVC rack system (Allentown Inc., Allentown, NJ) and placed on irradiated FF diet which contained 10% total fiber (wt/wt) composed of 5 fermentable fibers (inulin, pectin, short-chain FOS, RS-2 and RS-4; Supplementary Fig. 1, Supplementary Table 1). Inclusion rates of each fermentable fiber source were individually adjusted based on purity and ash content to achieve an effective inclusion rate of 2% of dietary fiber from each fiber source. One week later, GF mice were colonized with microbiota from one of the WLS fecal specimens (DonA, DonB, or DonC) by a single oral gavage of a fecal slurry or by cohousing. Slurries were prepared anaerobically by homogenizing ~200 mg of frozen human feces in 5 mL of pre-reduced Mega Media^35^ in an anaerobic chamber, and then were immediately used to gavage recipient mice using syringes flushed with anaerobic atmosphere. A subset of GF mice was colonized by cohousing together with mice that had been gavage-colonized with human feces 4 weeks prior. Cohousing is an effective strategy for colonizing germ-free mice and is similar to gavage in terms of microbiota colonization and phenotype transfer^56,57^. We were unable to detect differences in cecal microbial profiles, nor did we observe significant differences in phenotypes between cohoused mice and their gavage-colonized counterparts (Supplementary Fig. 2a, b, Supplementary Table 2). Therefore, we considered all mice within the same treatment group as biological replicates regardless of colonization method. Operating under the rationale that a diet with a greater diversity of fiber sources would promote colonization of more microbes, mice were maintained on the FF diet for an additional two weeks to allow colonization to stabilize before the dietary treatment phase. Upon dietary treatment, mice either continued the FF diet or were switched to the CC diet containing 10% cellulose (Supplementary Table 1), a non-fermentable fiber control. All experimental diets in this study were vacuum packed and irradiation-sterilized by the manufacturer. The FF diet and CC diet differed only in their fiber sources. Our experimental design (Supplementary Fig. 1) resulted in six treatment groups (three donors and two diets; *n* = 7–10 mice per treatment group), each of which were conducted in two separately-caged cohorts to account for cage effects. After 11 weeks of dietary treatment, mice were sacrificed at 20 weeks of age after 4 h of fasting.

### Atherosclerotic lesion analysis

Upon sacrifice, the heart was perfused with PBS buffer before being cut laterally at the mid heart and the ascending aorta to capture the aortic sinus. This tissue was embedded in OCT compound, frozen on dry ice, and stored at −80 °C until further processing. To characterize atherosclerotic plaques in the aortic sinus, the embedded tissue was sectioned on a cryostat (CM1950, Leica, Deer Park, IL) and collected on slides in 100 µm intervals, moving proximally from the base of the aortic root toward the ascending aorta. This resulted in slides containing eight equidistant sections (10 µm thickness) spanning 700 µm of the aortic sinus (0, 100, 200, 300, 400, 500, 600, 700 µm from the base of the aortic root). Formalin-fixed slides from each mouse were rinsed with 60% isopropanol for 1 min, stained for lipids using Oil Red O for 15 min, and counter-stained with hematoxylin for 1 min. Macrophage infiltration assessment of atherosclerotic plaques was conducted by incubating formalin-fixed slides (same sectioning pattern as above) overnight with macrophage antibodies (MOMA-2, 1:50; ab33451, Abcam, Cambridge, UK) followed by incubation with secondary antibodies (1:400; ab6733, Abcam) for 1 h and streptavidin horseradish peroxidase (1:500; P0397, Agilent, Santa Clara, CA) for 15 min. Sections were then washed with PBS and counterstained with DAB for 15 s and hematoxylin for 5 s. Images of all stained sections were digitally captured and then analyzed on ImageJ (National Institutes of Health, Bethesda, MD) to measure lipid-positive area, total plaque area, and MOMA-2 positive area. Plaque areas and lipid-positive areas for each mouse are expressed as averages across all eight sections. To calculate macrophage infiltration, the three sections with the largest visible lesions were selected from each mouse and their MOMA-2 positive area densities were averaged. One sample was lost during processing, so sample sizes for atherosclerosis characterization ranged from 7–10 samples per treatment group.

### Cecal short-chain fatty acids

SCFA levels in cecal contents were measured using headspace gas chromatography. Samples were prepared by adding 20–150 mg of frozen cecal contents to vials (Restek, Bellefonte, PA) containing *N* µL of water (where *N* equals 300 minus the mg of cecal content) along with 2 g of NaH_2_SO_4_ and 1 mL of chilled 60 µM 2-butanol as an internal standard. The preparations were immediately sealed in a GC sampling vial then allowed to sit overnight at RT. Standards for acetate, propionate, isobutyrate, butyrate, isovalerate, valerate and were combined at known concentrations (pH 7.0) and serially diluted to generate a standard curve. Vials were loaded into a HS-20 headspace sampler (Shimadzu, Columbia, OH), shaken for 20 min at 80 °C and injected onto an SH-Stabilwax 30 m column (227-36246-01, Shimadzu) connected to a flame ionization detector on a CG-2010 Plus GC (Shimadzu). Running conditions were as follows: the sample vial was equilibrated at 80 kPa for 3 min before injection; injection was performed using a 2 mL injection loop, a 12 s loading period with the transfer line at 150 °C, 1:15 split ratio, and a N_2_ column flow of 1.2 mL/min; the column temperature was maintained at 40 °C for 2 min and then increased to 200 °C at a rate of 20 °C/min, held for 2 min, and then reduced to 120 °C (−20 °C/min), reduced to 40 °C (−40 °C/min) and held at 40 °C for 1 min. Areas under the curve for each target compound were calculated with Shimadzu Lab Solution software (version 5.92) and normalized by the sample mass and the dilution factor and converted to μmol·g^−1^ using a standard curve.

### Plasma triglyceride and cholesterol measurements

Blood was collected from mice while under isoflurane-induced anesthesia by cardiac puncture using an EDTA-rinsed syringe. Blood cells were separated by centrifugation then plasma was collected and stored at −80 °C. Plasma levels of triglycerides, total cholesterol, and high-density lipoprotein (HDL) cholesterol, were measured using commercially available colorimetric assay kits from Waco Diagnostics (Cat. No. 994-02891, 99902601, 997-01301, respectively; Fujifilm, Tokyo, Japan) in accordance with manufacturer’s instructions.

### Quantitative real-time PCR

Total RNA was extracted from frozen aorta using TRIzol reagent (Invitrogen/Thermo Fisher Scientific, Waltham, MA) with 2-min of bead-beating (BioSpec Products, Barlesville, OK) at RT in tubes containing 1.0 g of 1 mm diameter zirconium beads (BioSpec Products) and cleaned with the Qiagen RNeasy mini kit (Qiagen, Hilden, Germany). Template cDNA was synthesized using 125 ng of purified RNA in 20 µL reaction volumes. cDNA was diluted 1:1 with water then 1 µL was mixed with SYBR qPCR Mastermix (Bio-Rad, Hercules, CA) and combined with the appropriate primers (400 nM) and water for a total reaction volume of 10 µL. A list of primers is shown in Supplementary Table 3. The cycling protocol was performed using Mastercycler® nexus (Eppendorf, Hamburg, Germany) as follows: 30 s at 95 ˚C, followed by 35 cycles of 10 s at 95 ˚C, 30 s at 60 ˚C. A melt curve was conducted from 65 ˚C to 95 ˚C at increments of 0.5 ˚C at 5 sec/step. All reactions were run in duplicate and delta-delta-Ct values were calculated relative to the endogenous control (*Gapdh*).

### 16S rRNA gene sequencing

DNA was extracted from all mouse cecal content and feces as well as human fecal slurries using a phenol-chloroform extraction method that included a bead-beating step^58^. 16S rRNA gene (V4) amplification was done by PCR involving unique barcodes (8-bp) both on the forward and reverse primers what are fused to Illumina adapters^59^. The V4 amplicons from each sample were combined and submitted for sequencing on an Illumina MiSeq run (2 × 250 bp, Illumina, San Diego, CA) at the University of Wisconsin, Madison Biotechnology Center’s DNA Sequencing Facility. Samples with less than 20% of the average sample read count were excluded, resulting in the removal of four samples from further analysis. The remaining samples ranged from 33,869 to 133,538 paired-end reads with an average of 77,704 paired-end reads per sample. Qiime2 (version 2019.10) was used to generate amplicon sequence variant (ASV) tables and taxonomy tables from the 16S rRNA reads. Demultiplexed reads were trimmed and filtered for quality with the Qiime2 DADA2 plug-in^60^. ASVs were annotated to the genus level with SILVA reference database^61^ (version 132) using Naïve Bayes classifier in Qiime2. All subsequent analysis were conducted in R. ASV-level feature counts normalized by converting to relative abundance. ASVs were filtered to include only those with at least 0.0001 average relative abundance across all cecal samples. For genus-level analysis, a cutoff was set at 0.0005 average relative abundance across all samples. These cutoffs were also applied to the fecal sample ASV and genera profiles (human inoculum fecal slurries and pre-dietary treatment mouse feces). Engraftment efficiency was calculated as the number of features that were detected in the pre-treatment mouse feces of at least one animal per donor- or donor/diet-group divided by the number of features detected in the donor fecal slurry. Positive detection was defined as any feature (ASV or genus) that was found to be greater than 0.0001 relative abundance in a given sample.

### Shotgun sequencing

Genomic DNA isolated from the cecal contents of 5 mice per treatment group was used for metagenomic analysis. Libraries were prepared using the Illumina TruSeq PCR-free kit following vendor protocols and sequenced at the University of Wisconsin Biotechnology Center’s DNA Sequencing Facility. All samples were run on a single NovaSeq6000 2 × 150 S4-Flowcell lane. The resulting sequences were trimmed for quality using Trimmomatic (version 0–39) and then aligned against reference host genomes (*Mus musculus* GRCm38_Rel98) with bowtie2 (version 2.3.4) to remove host reads (average host alignment rate was 5.3%) leaving only high-quality, non-host reads. Cleaning ultimately resulted in an average of 29.4 million paired-end reads per sample.

#### Functional annotation

Reads remaining after trimming and removal of host sequences were concatenated into a single fastq file and fed into HUMAnN3 (version 3.0.0.alpha.4) for functional annotation. This resulted in a UniRef90^62^ gene family abundance table in reads per kilobase, and a relative abundance table of microbial taxa for each mouse. The UniRef90 gene family abundance tables were converted to KO counts-per-million (CPM) abundance tables with the human_regroup_table and human_renorm_table functions. Differential abundance analysis was conducted on KOs that were present in at least 25% of samples.

#### CAZyme annotation

CAZyme profiles of each sample were predicted using run_dbcan (version 2.0.11). We assembled cleaned (trimmed, host-free) reads into contigs with metaSPAdes (version 3.14.0) with multiple k-mer sizes (metaspades.py -k 21, 33, 55, 77). Contigs shorter than 500 bp were discarded from further processing. Open reading frames (ORFs; i.e., microbial metagenes), were predicted from assembled contigs via Prodigal (version 2.6.3) using Hidden Markov Model (HMM) with default parameters. All predicted genes shorter than 100 bp were discarded from further processing. Nucleotide ORF sequences were converted amino acid sequences and were used as input for run_dbcan (version 2.0.11) to predict CAZyme profiles. CAZyme annotation was accepted if an ORF was annotated by >= 2 tools (DIAMOND, HMMER, Hotpep). This resulted in a table indicating the presence or absence of each CAZyme family in the CAZyme database. To estimate CAZyme abundance, each CAZyme family was assigned a count-per-million (CPM) value of its associated ORF as predicted by Prodigal. If multiple CAZymes were predicted from the same ORF, they were all assigned the ORF’s CPM value.

### Microbiome analysis

PCoA plots and diversity measures were generated using 16S rRNA ASV profiles with the phyloseq (version 1.40.0) package in R. All pairwise PERMANOVA tests were conducted between dietary groups within each donor group using the pairwiseAdonis (version 0.4) R package with 9999 permutations. Feature-level differential abundance analysis of 16S rRNA amplicon taxonomy, shotgun metagenomic KO abundances and shotgun metagenomic CAZyme abundances were conducted using the MaAsLin 2 function within the MaAsLin 2 (version 1.10.0) R package with default settings^41^. To assess KO pathway enrichment, sets of KOs that were significantly upregulated by FF feeding (MaAsLin 2 differential abundance, adjusted *P* < 0.1) were generated for each donor group and used as input for the PerformKOEnrichAnalysis_KO01100 function within the MicrobiomeAnalyistR (version 0.0.0.9000) package in R with default parameters. This resulted in a list of KEGG pathways that were significantly (*P* < 0.05) overrepresented in FF-fed mice within each donor group. CAZymes families were considered to be highly differential if they belonged to the top 10% of CAZymes ordered by MaAsLin 2 effect size within each donor group, regardless of direction (absolute value of effect size).

### Statistical analysis

Unless otherwise noted, comparisons of means were conducted using a two-sided Wilcoxon rank-sum test between dietary groups within each donor group. Equal variance was determined for all means tests (Levene’s test, *P* > 0.05) except comparisons of atherosclerotic plaques (size, lipid content, macrophage infiltration) and cecal SCFA levels which were found to have significantly different variances between diets (Levene’s test, *P* < 0.05). Correlations between CAZymes and cecal SCFA levels were conducted using all mice to calculate Spearman’s rank correlation coefficient. *P*-value adjustment for PERMANOVA was done using the Bonferroni method, while all other adjusted *P*-values were calculated using the Benjamini–Hochberg method.

### Reporting summary

Further information on research design is available in the Nature Research Reporting Summary linked to this article.

## Supplementary information

Supplementary material

Reporting Summary

## Data Availability

Sequencing data reported in this study is available at the European Nucleotide Archive (ENA) under the study accession number PRJEB58699.

## Code availability

The code used in this study is available upon request.
