# Supplementary material for: Dissecting the impact of dietary fiber type on atherosclerosis in mice colonized with different gut microbial communities
Source: NPJ Biofilms Microbiomes. 2023 Jun 3;9:31. doi: 10.1038/s41522-023-00402-7 (PMC10239454; doi:10.1038/s41522-023-00402-7)
Supplement: Supplementary file 1 — Supplementary material [file 41522_2023_402_MOESM1_ESM.pdf]

Supplementary Figures and Tables

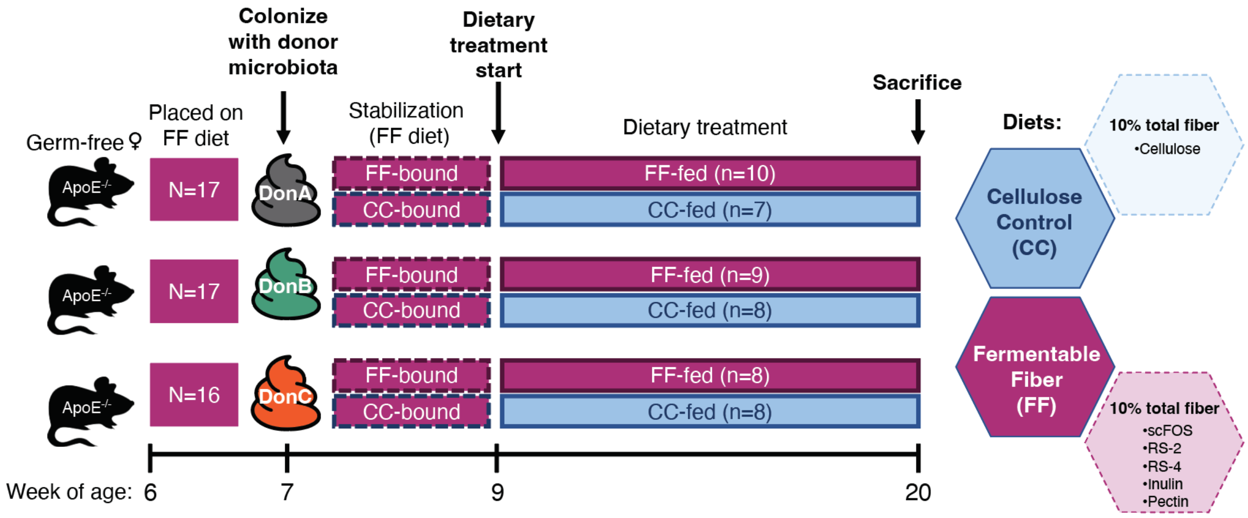

Supplementary Fig. 1 Schematic of the experimental design used in this study.

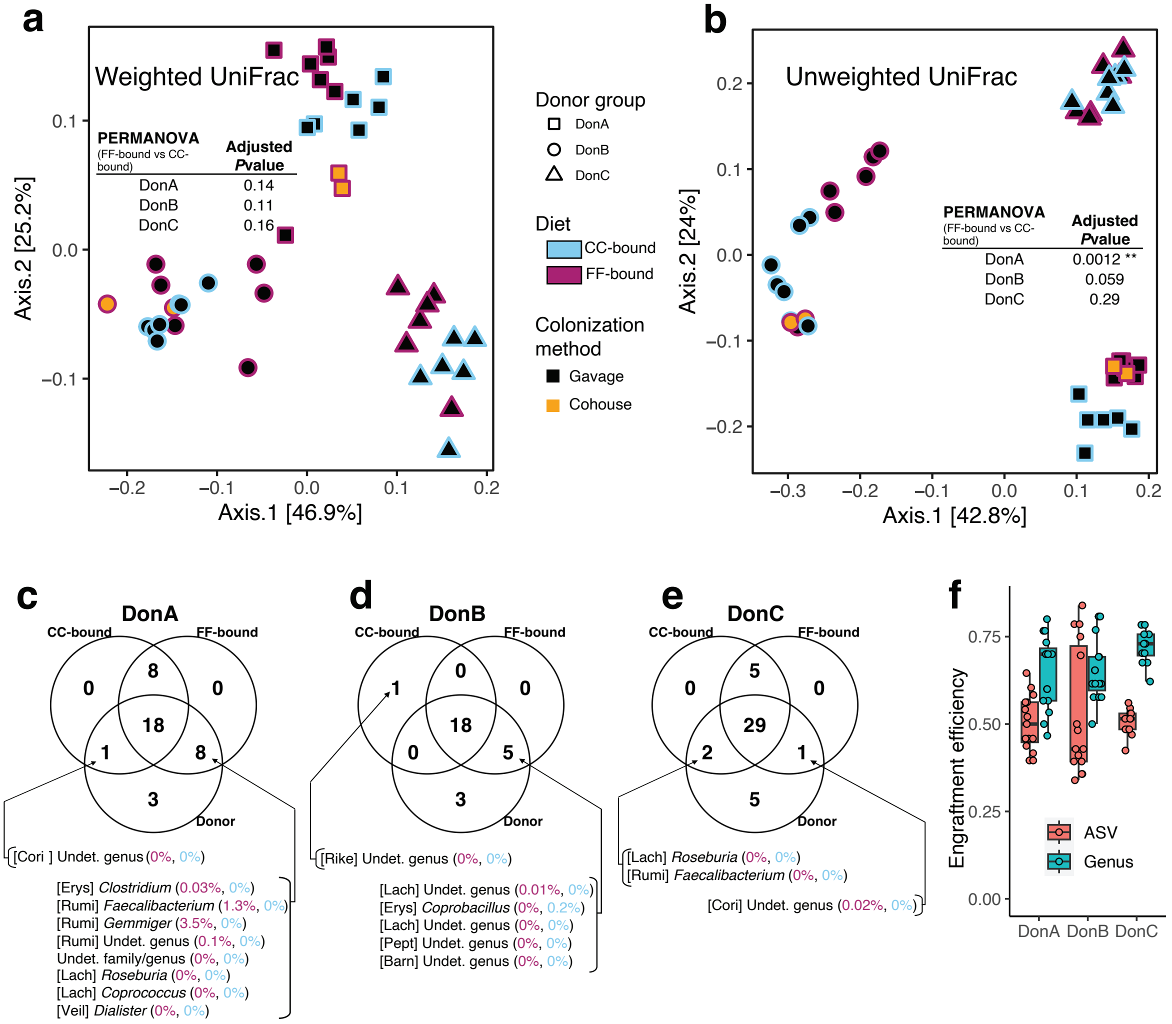

Supplementary Fig. 2 Engraftment of genera from each donor fecal sample into recipient mice prior to dietary treatment**.** Principal coordinate analysis (PCoA) of pre-treatment fecal samples using weighted (**a**) and unweighted (**b**) UniFrac distances. Colonization method is denoted by the inner color of each point (black = gavage, orange = cohouse) and assigned diet group is denoted by the outer color (FF-bound = magenta, CC-bound = blue). **c-e** Venn diagrams of the genera detected in the donor fecal sample and in at least one mouse belonging to the CC-bound group or the FF-bound group of mice two weeks after colonization and prior to beginning the dietary treatment phase. The genera listed below the Venn diagrams are denoted by their family (in brackets) and their group-average abundance in the cecal content of mice belong to the CC-fed group (blue text) or the FF-fed group (magenta text) at the end of the study after dietary treatment. Undet. = undetermined. **f** Engraftment efficiency for each mouse is expressed at the of genus level (teal) or at the ASVs level (salmon) two weeks after inoculation, but prior to dietary treatment.

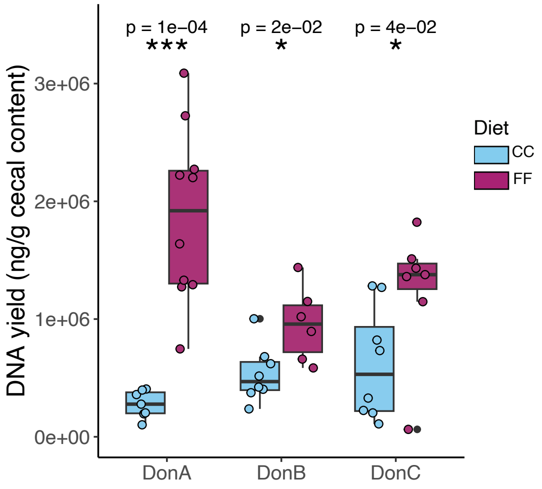

Supplementary Fig. 3 Fecal DNA yields per gram of cecal content obtained from mice colonized with human fecal communities DonA, DonB, and DonC and fed either fermentable fiber diet (FF, magenta), or a cellulose control diet (CC, blue). Box and whisker plots denote the interquartile range, median, and spread of points within 1.5 times the interquartile range along with individual data points. Comparisons of means between diets within each donor group (*n* = 7-10/diet/donor group) were conducted using a Wilcoxon test, * *P* <0.05, *** *P* <0.001.

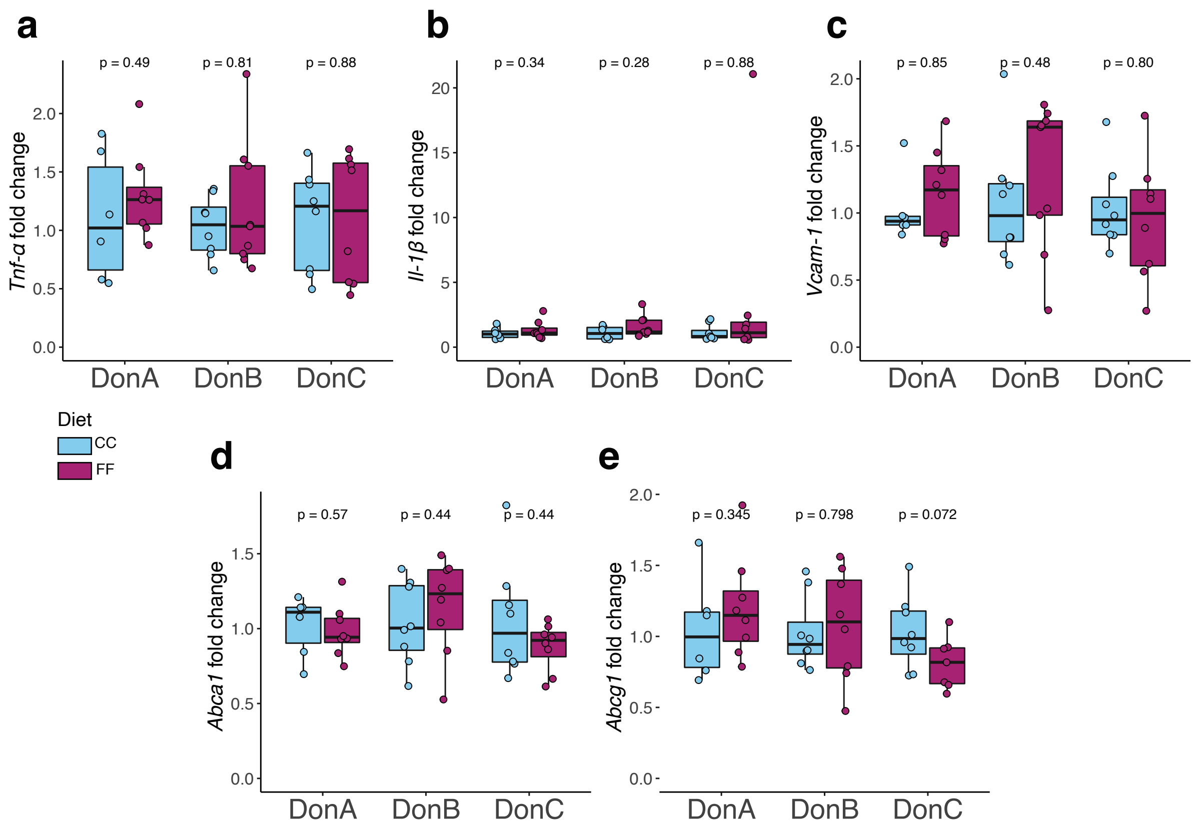

Supplementary Fig. 4 Quantitative RT-PCR measurements of inflammatory markers and cholesterol efflux genes in aortic samples. **a-c** Log_2 fold-changes of mRNA abundance for inflammatory markers, and **d,e** genes encoding subunits of key cholesterol transporters between mice consuming FF and CC diets. All fold-changes are expressed relative to the donor-matched CC-fed group and were calculated using *Gapdh* as a reference gene. Box and whisker plots denote the interquartile range, median, and spread of points within 1.5 times the interquartile range along with individual data points. Comparisons of means between diets within each donor group were conducted using a Wilcoxon test.

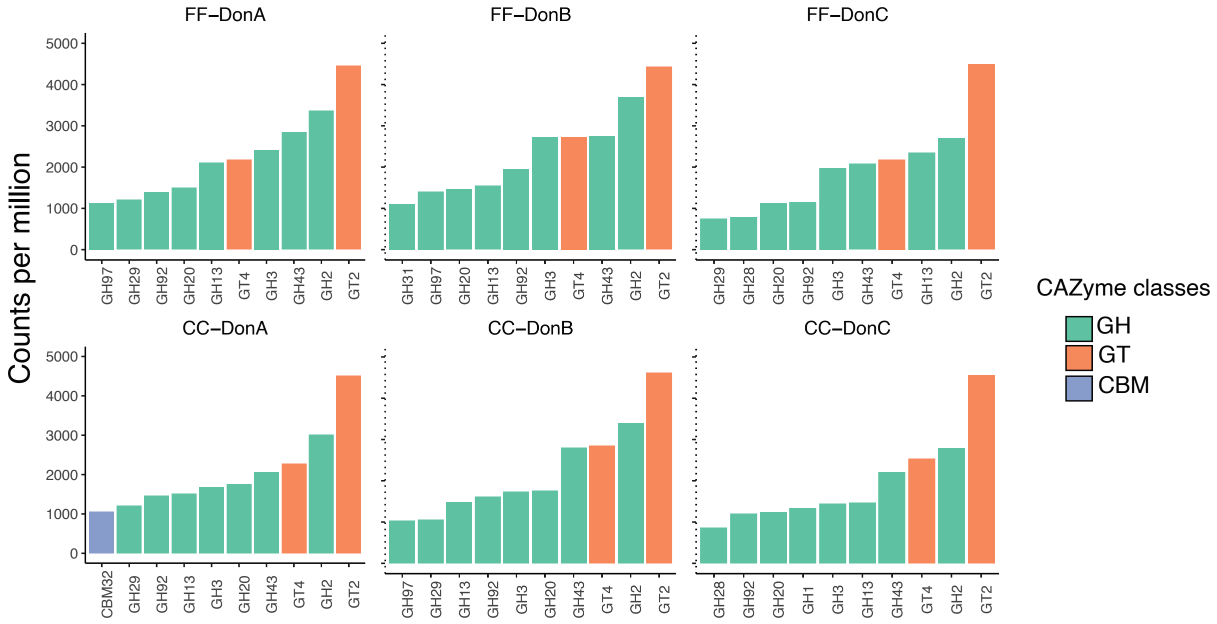

Supplementary Fig. 5 Average (mean) CPM of the top 10 most abundant CAZyme families within each diet-donor group (FF top, CC bottom). CAZyme classes are indicated by color (Glycoside hydrolase = GH; glycosyltransferases = GT; carbohydrate binding modules = CBM). Values are expressed in counts per million (CPM).

Supplementary Table 1. Composition of diets. Macronutrient composition and ingredient list of the fermentable fiber and cellulose control diets.

**Macronutrient**	**Cellulose diet (CC)**	**Fermentable fiber diet (FF)**
Protein, % by weight	17.7	17.7
Carbohydrate, % by weight	48.4	47.9
Fat, % by weight	15.2	15.2
Protein, % Kcal from	17.7	17.7
Carbohydrate, % Kcal from	48.2	48
Fat, % Kcal from	34.1	34.3
Kcal/g	4	4
Ingredient	*(g/Kg)*	
Casein	200	200
L-Cystine	3	3
AMIOCA	261.22	236.02
Maltodextrin	132	132
Sucrose	100	100
Cellulose	100	0
Soybean Oil	75	75
Lard	75	75
Mineral Mix, AIN-93G-MX	35	35
Vitamin Mix, AIN-93-VX	16	16
Choline Bitartrate	2.75	2.75
TBHQ, antioxidant	0.03	0.03
Inulin	0	23.4
Fructooligosaccharide (FOS)	0	21.5
HI-MAIZE 260 (Resistant starch type 2)	0	33.3
Resistant wheat starch (Resistant starch type 4)	0	23.5
Pectin	0	23.5

Supplementary Table 2. Comparison of phenotypes between gavage- and cohouse-colonozed mice . All mice were colonized with human microbiota via gavage except subset of mice within the FF-fed DonA and DonB groups which were colonized by cohousing with 2 gavage-colonized mice. Statistical comparison of terminal phenotypes between gavage-colonized mice and their cohouse-colonized counterparts within each affected treatment group.

		P -value	
**Phenotype**	**Statistical test**	**DonA-FF**	**DonB-FF**
Cecal 16S rRNA community profiles (Unweighted UniFrac distances)	PERMANOVA	0.43	0.10
Cecal 16S rRNA community profiles (Weighted UniFrac distances)	PERMANOVA	0.10	0.30
Plaque area	Wilcoxon	0.11	0.70
Oil-Red-O area	Wilcoxon	0.48	0.70
MOMA-2 density	Wilcoxon	1.00	1.00
Cecal acetate	Wilcoxon	0.35	0.70
Cecal propionate	Wilcoxon	1.00	1.00
Cecal isobutyrate	Wilcoxon	0.48	0.10
Cecal butyrate	Wilcoxon	0.61	0.40
Cecal isovalerate	Wilcoxon	0.91	0.10
Plasma triglycerides	Wilcoxon	0.61	1.00
Plasma total cholesterol	Wilcoxon	0.26	0.20
Plasma HDL cholesterol	Wilcoxon	0.17	0.20
Tnf-α fold-change	Wilcoxon	0.79	1.00
Il1-**β** fold-change	Wilcoxon	0.79	0.40
Vcam-1 fold-change	Wilcoxon	0.25	1.00
Abca1 fold-change	Wilcoxon	0.79	0.40
Abcg1 fold-change	Wilcoxon	1.00	0.40

Supplementary Table 3. Primers used for qPCR assays.
Forward primer sequences for each target gene are nedoted by "-F" and reverse sequences are denoted by "-R".

Primer name	Sequence
Abca1-F	GGTTTGGAGATGGTTATACAATAGTTGT
Abca1-R	CCCGGAAACGCAAGTCC
Abcg1-F	TCACCCAGTTCTGCATCCTCTT
Abcg1-R	GCAGATGTGTCAGGACCGAGT
Tnfα-F	ATGAGCACAGAAAGCATGATC
Tnfα-R	TACAGGCTTGTCACTCGAATT
Il-6-F	CCACTTCACAAGTCGGAGGCTTA
Il-6-R	GCAAGTGCATCATCGTTGTTCATAC
Vcam1-F	TGCCGGCATATACGAGTGTGA
Vcam1-R	CCCGATGGCAGGTATTACCAAG
Gapdh-F	TGTGTCCGTGGATCTGA
Gapdh-R	CCTGCTTCACCTTCTTGA
