# Supplementary material for: Dissecting the impact of dietary fiber type on atherosclerosis in mice colonized with different gut microbial communities
Source: NPJ Biofilms Microbiomes. 2023 Jun 3;9:31. doi: 10.1038/s41522-023-00402-7 (PMC10239454; doi:10.1038/s41522-023-00402-7)
Supplement: Supplementary file 2 — Reporting Summary [file 41522_2023_402_MOESM2_ESM.pdf]

nature portfolio

Corresponding author(s): Federico E. Rey

Last updated by author(s): Mar 27, 2023

Reporting Summary

Nature Portfolio wishes to improve the reproducibility of the work that we publish. This form provides structure for consistency and transparency in reporting. For further information on Nature Portfolio policies, see our Editorial Policies and the Editorial Policy Checklist.

Statistics

For all statistical analyses, confirm that the following items are present in the figure legend, table legend, main text, or Methods section.

n/a	Confirmed	
☐	☒	The exact sample size (*n*) for each experimental group/condition, given as a discrete number and unit of measurement
☐	☒	A statement on whether measurements were taken from distinct samples or whether the same sample was measured repeatedly
☐	☒	The statistical test(s) used AND whether they are one- or two-sided<br>*Only common tests should be described solely by name; describe more complex techniques in the Methods section.*
☐	☒	A description of all covariates tested
☐	☒	A description of any assumptions or corrections, such as tests of normality and adjustment for multiple comparisons
☐	☒	A full description of the statistical parameters including central tendency (e.g. means) or other basic estimates (e.g. regression coefficient) AND variation (e.g. standard deviation) or associated estimates of uncertainty (e.g. confidence intervals)
☐	☒	For null hypothesis testing, the test statistic (e.g. *F*, *t*, *r*) with confidence intervals, effect sizes, degrees of freedom and *P* value noted<br>*Give P values as exact values whenever suitable.*
☒	☐	For Bayesian analysis, information on the choice of priors and Markov chain Monte Carlo settings
☐	☒	For hierarchical and complex designs, identification of the appropriate level for tests and full reporting of outcomes
☐	☒	Estimates of effect sizes (e.g. Cohen's *d*, Pearson's *r*), indicating how they were calculated

Our web collection on statistics for biologists contains articles on many of the points above.

Software and code

Policy information about availability of computer code

Data collection	Measurements for the plasma: total cholesterol, TAG, HDL-cholesterol; from aortic sinus: plaque area, MPMA-2 area, lipid area; from aorta: mRNA expression by RT-qPCR; from cecal content: 16S rRNA amplicon sequences, shotgun metagenomic sequences, SCFA levels, DNA content.
Data analysis	16S rRNA V4 amplicons were sequencesd using an Illumina MiSeq (2x250 bp, Illumina, San Diego, CA) at the University of Wisconsin, Madison Biotechnology Center's Sequencing Core. Qiime2 (version 2019.10) was used to generate amplicon sequence variant (ASV) tables and taxonomy tables from the 16S rRNA reads. Reads were trimmed and filtered for quality with the Qiime2 DADA2 plug-in. ASVs were annotated to the genus level with SILVA_132_V4 reference database using Naïve Bayes classifier in Qiime2. Shotgun reads were generated on a NovaSeq6000 2x150 S4-Flowcell lane. The resulting sequences were trimmed for quality using Trimmomatic (version 0-39) and then aligned against reference host genomes (Mus musculus GRCm38_Rel98) with bowtie2 (version 2.3.4) to remove host reads. Clean reads were annotated using HUMAnN3 (version 3.0.0.alpha.4). The UniRef90 gene family abundance tables were converted to KO counts per million (CPM) abundance tables with the human_regroup_table and human_renorm_table functions in HUMAnN3. CAZyme profiles of cleaned shotgun reads were predicted using run_dbcan (version 2.0.11). Assembled cleaned (trimmed, host-free) reads assembled into contigs with metaSPAdes (version 3.14.0) with multiple k-mer sizes (metaspades.py -k 21, 33, 55, 77). Open reading frames (ORFs; i.e., microbial metagenes), were predicted from assembled contigs via Prodigal70 (version 2.6.3) using Hidden Markov Model (HMM) with default parameters and fed into run_dbcan. PCoA plots and diversity measures were generated using 16S rRNA ASV profiles with the phyloseq (version 1.40.0) package in R. All pairwise PERMANOVA tests were conducted between dietary groups within each donor group using the pairwiseAdonis (version 0.4) R package with 9999 permutations. Feature-level differential abundance analysis of 16S rRNA amplicon taxonomy, shotgun metagenomic KO abundances and shotgun metagenomic CAZyme abundances were conducted using the MaAsLin 2 function within the MaAsLin 2 (version 1.10.0) R package with default settings. KO pathway enrichment was conducted using the

PerformKOEnrichAnalysis_KO01100 function within the MicrobiomeAnalystR (version 0.0.0.9000) package in R with default parameters. The code used in this study is available upon request.

For manuscripts utilizing custom algorithms or software that are central to the research but not yet described in published literature, software must be made available to editors and reviewers. We strongly encourage code deposition in a community repository (e.g. GitHub). See the Nature Portfolio guidelines for submitting code & software for further information.

Data

Policy information about availability of data

All manuscripts must include a data availability statement. This statement should provide the following information, where applicable:

- Accession codes, unique identifiers, or web links for publicly available datasets
- A description of any restrictions on data availability
- For clinical datasets or third party data, please ensure that the statement adheres to our policy

Sequencing data reported in this study is available at the European Nucleotide Archive (ENA) under the study accession number PRJEB58699.

Human research participants

Policy information about studies involving human research participants and Sex and Gender in Research.

Reporting on sex and gender	Neither sex nor gender were considered, nor was sex or gender-based analysis performed on the human samples in this study.
Population characteristics	We used 3 fecal samples from participants in the Wisconsin Longitudinal Study (WLS) (PMID: 28444239). WLS is a cohort of Wisconsin residents who graduated high school in 1957 (n=10,317). The fecal samples were used to colonize germ-free mice in this study.
Recruitment	The recruitment of participants of the WLS is described in Herd, 2018 (PMID: 28444239).
Ethics oversight	WLS data collection was approved by the UW-Madison Internal Review Board (2014-1066, 2015-0955).

Note that full information on the approval of the study protocol must also be provided in the manuscript.

Field-specific reporting

Please select the one below that is the best fit for your research. If you are not sure, read the appropriate sections before making your selection.

☒ Life sciences ☐ Behavioural & social sciences ☐ Ecological, evolutionary & environmental sciences

For a reference copy of the document with all sections, see nature.com/documents/nr-reporting-summary-flat.pdf

Life sciences study design

All studies must disclose on these points even when the disclosure is negative.

Sample size	A target sample size of 8 per group was chosen based off of atherosclerosis data from previous experiments with the same mouse strain on a similar diet. The final sample size was 7-10 mice per group.
Data exclusions	One mouse was excluded from atherosclerosis analysis because the sample was damaged. Three mice were excluded from 16S rRNA sequencing analysis due to low read count. These mice were also excluded from MOMA-2 staining.
Replication	Biological replicates (7-10 replicates per treatment group). 2-3 technical replicates per sample were used in all colorimetric and RT-qPCR assays.
Randomization	Mice were randomized before placing into treatment groups. All tissue samples were randomized prior to processing and assaying.
Blinding	All samples received an non-descriptive sample ID which did not contain information about the sample's treatment group. This ID was used throughout the study. All samples were blinded to the researcher for aortic sinus processing and staining using a secondary, randomized index. Unblinding the samples required the use of a key at all points throughout the study. This key was available to the researchers and was used in the R code to generate figures.

Behavioural & social sciences study design

All studies must disclose on these points even when the disclosure is negative.

Study description	*Briefly describe the study type including whether data are quantitative, qualitative, or mixed-methods (e.g. qualitative cross-sectional, quantitative experimental, mixed-methods case study).*
Research sample	*State the research sample (e.g. Harvard university undergraduates, villagers in rural India) and provide relevant demographic information (e.g. age, sex) and indicate whether the sample is representative. Provide a rationale for the study sample chosen. For studies involving existing datasets, please describe the dataset and source.*
Sampling strategy	*Describe the sampling procedure (e.g. random, snowball, stratified, convenience). Describe the statistical methods that were used to predetermine sample size OR if no sample-size calculation was performed, describe how sample sizes were chosen and provide a rationale for why these sample sizes are sufficient. For qualitative data, please indicate whether data saturation was considered, and what criteria were used to decide that no further sampling was needed.*
Data collection	*Provide details about the data collection procedure, including the instruments or devices used to record the data (e.g. pen and paper, computer, eye tracker, video or audio equipment) whether anyone was present besides the participant(s) and the researcher, and whether the researcher was blind to experimental condition and/or the study hypothesis during data collection.*
Timing	*Indicate the start and stop dates of data collection. If there is a gap between collection periods, state the dates for each sample cohort.*
Data exclusions	*If no data were excluded from the analyses, state so OR if data were excluded, provide the exact number of exclusions and the rationale behind them, indicating whether exclusion criteria were pre-established.*
Non-participation	*State how many participants dropped out/declined participation and the reason(s) given OR provide response rate OR state that no participants dropped out/declined participation.*
Randomization	*If participants were not allocated into experimental groups, state so OR describe how participants were allocated to groups, and if allocation was not random, describe how covariates were controlled.*

Ecological, evolutionary & environmental sciences study design

All studies must disclose on these points even when the disclosure is negative.

Study description	*Briefly describe the study. For quantitative data include treatment factors and interactions, design structure (e.g. factorial, nested, hierarchical), nature and number of experimental units and replicates.*
Research sample	*Describe the research sample (e.g. a group of tagged Passer domesticus, all Stenocereus thurberi within Organ Pipe Cactus National Monument), and provide a rationale for the sample choice. When relevant, describe the organism taxa, source, sex, age range and any manipulations. State what population the sample is meant to represent when applicable. For studies involving existing datasets, describe the data and its source.*
Sampling strategy	*Note the sampling procedure. Describe the statistical methods that were used to predetermine sample size OR if no sample-size calculation was performed, describe how sample sizes were chosen and provide a rationale for why these sample sizes are sufficient.*
Data collection	*Describe the data collection procedure, including who recorded the data and how.*
Timing and spatial scale	*Indicate the start and stop dates of data collection, noting the frequency and periodicity of sampling and providing a rationale for these choices. If there is a gap between collection periods, state the dates for each sample cohort. Specify the spatial scale from which the data are taken*
Data exclusions	*If no data were excluded from the analyses, state so OR if data were excluded, describe the exclusions and the rationale behind them, indicating whether exclusion criteria were pre-established.*
Reproducibility	*Describe the measures taken to verify the reproducibility of experimental findings. For each experiment, note whether any attempts to repeat the experiment failed OR state that all attempts to repeat the experiment were successful.*
Randomization	*Describe how samples/organisms/participants were allocated into groups. If allocation was not random, describe how covariates were controlled. If this is not relevant to your study, explain why.*
Blinding	*Describe the extent of blinding used during data acquisition and analysis. If blinding was not possible, describe why OR explain why blinding was not relevant to your study.*

Did the study involve field work? ☐ Yes ☒ No

Reporting for specific materials, systems and methods

We require information from authors about some types of materials, experimental systems and methods used in many studies. Here, indicate whether each material, system or method listed is relevant to your study. If you are not sure if a list item applies to your research, read the appropriate section before selecting a response.

Materials & experimental systems

n/a	Involved in the study	
☐	☒	Antibodies
☒	☐	Eukaryotic cell lines
☒	☐	Palaeontology and archaeology
☐	☒	Animals and other organisms
☒	☐	Clinical data
☒	☐	Dual use research of concern

Methods

n/a	Involved in the study	
☒	☐	ChIP-seq
☒	☐	Flow cytometry
☒	☐	MRI-based neuroimaging

Antibodies

Antibodies used	MOMA-2 (Anti-Monocyte + Macrophage antibody): Abcam, Cat#;ab33451, Clone; MOMA-2, 1:50, Specificity; the antibody recognizes an intracellular antigen of mouse macrophages and monocytes. Species reactivity; Mouse; (lot number not available)<br><br>Rabbit Anti-Rat IgG H&L (Biotin): Abcam, Cat#;ab6733, 1:400, Polyclonal, Species reactivity; Rat; (lot number not available)
Validation	These antibodies are validated regularly my the manufacturer.<br><br>For MOMA-2, the manufacturer says: "This antibody recognises an intracellular antigen of mouse macrophages and monocytes. It reacts strongly with macrophages in lymphoid organs in all mouse strains tested. Staining may also be seen on the epithelium of mucosa."<br><br>This combination of antibodies was also used in Kasahara, 2018 (PMID: 30397344).

Animals and other research organisms

Policy information about studies involving animals; ARRIVE guidelines recommended for reporting animal research, and Sex and Gender in Research

Laboratory animals	Germ-free (GF) ApoE-/- mice (derived from Apoe-tm1Unc, MGI:1857129).
Wild animals	No wild animals were used in this study.
Reporting on sex	Only female mice were used in this study. Female ApoE-/- mice develop atherosclerotic lesions faster than males on a low cholesterol diet (PMID: 10488957) and were chosen to maximize resource use efficiency.
Field-collected samples	This study did not involve field samples.
Ethics oversight	This protocols used in this study were approved by the UW-Madison Animal Care and Use Committee under protocol M005599.

Note that full information on the approval of the study protocol must also be provided in the manuscript.
